# ERRATUM: Evaluation of Programs of Infection Control related to Healthcare Assistance in Hospitals

**DOI:** 10.1590/1980-220X-REEUSP-2024-ER09en

**Published:** 2024-09-30

In the article “Evaluation of Programs of Infection Control related to Healthcare Assistance in Hospitals”, with the DOI: https://doi.org/10.1590/S0080-623420150000700010, published by the journal: “Revista da Escola de Enfermagem da USP, volume 49 (spe) of 2015, p. 65–72, on page 72:

**Where it was written:**

## Financial support

Fundação de Amparo à Pesquisa do Estado de São Paulo (FAPESP).

**Now read:**

## Financial support

Fundação de Amparo à Pesquisa do Estado de São Paulo (FAPESP). This study was financed in part by the Coordenação de Aperfeiçoamento de Pessoal de Nível Superior – Brasil (CAPES) – Finance Code 001.

