# Supplementary material for: ERRATUM: Evaluation of Programs of Infection Control related to Healthcare Assistance in Hospitals
Source: Rev Esc Enferm USP. 2024 Sep 30;58:e2024ER09. doi: 10.1590/1980-220X-REEUSP-2024-ER09en (PMC11468630; doi:10.1590/1980-220X-REEUSP-2024-ER09en)
Supplement: Translation

# Translation: ERRATA: Avaliação de Programas de Controle de Infecção relacionada a Assistência à Saúde de Hospitais

No artigo “Avaliação de Programas de Controle de Infecção relacionada a Assistência à Saúde de Hospitais”, com número de: DOI: https://doi.org/10.1590/S0080-623420150000700010, publicado na Revista da Escola de Enfermagem da USP, volume 49 (Esp) de 2015, p. 65–73, na página 73:

**Onde se lia:**

## Apoio financeiro

Fundação de Amparo à Pesquisa do Estado de São Paulo (FAPESP).

**Leia-se:**

## Apoio Finaceiro

Fundação de Amparo à Pesquisa do Estado de São Paulo (FAPESP). O presente trabalho foi realizado com apoio da Coordenação de Aperfeiçoamento de Pessoal de Nível Superior – Brasil (CAPES) – Código de Financiamento 001.
